# Molecular vibrational trapping revisited: a case study with D2+

**DOI:** 10.1038/srep31871

**Published:** 2016-08-23

**Authors:** Péter Badankó, Gábor J. Halász, Ágnes Vibók

**Affiliations:** 1 Department of Theoretical Physics, University of Debrecen, PO Box 5, H-4010, Debrecen, Hungary; 2 Department of Information Technology, University of Debrecen, PO Box 12, H-4010, Debrecen, Hungary; 3 ELI-ALPS, ELI-HU Non-Profit Ltd, Dugonics ter 13, H-6720, Szeged, Hungary

## Abstract

The present theoretical study is concerned with the vibrational trapping or bond hardening, which is a well-known phenomenon predicted by a dressed state representation of small molecules like 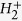 and 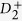
in an intense laser field. This phenomenon is associated with a condition where the energy of the light induced, vibrational level coincides with one of the vibrational levels on the field-free potential curve, which at the same time maximizes the wave function overlap between these two levels. One-dimensional numerical simulations were performed to investigate this phenomenon in a more quantitative way than has been done previously by calculating the photodissociation probability of 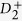 for a wide range of photon energy. The obtained results undoubtedly show that the nodal structure of the field-free vibrational wave functions plays a decisive role in the vibrational trapping, in addition to the current understanding of this phenomenon.

Understanding the behavior of atoms and molecules in a strong laser field is an intensively studied area and there are numerous experimental and theoretical investigations which have uncovered and explored several new phenomena of light-matter interactions including high harmonic generation, above threshold ionization or dissociation^1^,^2^,^3^,^4^. The bond softening and the bond hardening effects^5^,^6^,^7^,^8^,^9^,^10^,^11^,^12^,^13^,^14^,^15^,^16^,^17^,^18^,^19^,^20^,^21^,^22^,^23^,^24^ are similar phenomena which are often visualized during the photodissociation or photofragmentation processes of diatomic or polyatomic molecules. The mechanism of bond softening is well known. It was first discovered experimentally in the dissociation spectra of the 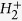 and 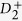 ions by Bucksbaum and coworkers^11^,^12^,^13^ and can easily be understood by the illustrative Floquet’s picture^25^,^26^ or dressed state representation, which is often used to explain various strong field physics phenomena. The Floquet Hamiltonian for the net absorption of one photon can be represented by a 2 × 2 matrix, which explicitly includes the light-matter interaction. The change of the nuclear dynamics, due to the laser field, can be visualized as arising from “light-induced” or “adiabatic” molecular potentials (LIP)^27^. The molecular potential deforms due to strong radiative coupling and results in a strongly enhanced dissociation rate. The adiabatic curves show that as the laser intensity increases, the dissociation barrier moves to lower vibrational levels, leading to a noticeable growth of the dissociation probability. Bond hardening, which is often called molecular stabilization or vibrational trapping is the opposite effect with the same origins. Despite numerous experimental and theoretical works that have been performed in order to gain a better qualitative understanding of the mechanism behind this phenomenon^5^,^6^,^7^,^8^,^9^,^10^,^11^,^12^,^13^,^14^,^15^,^28^,^29^,^30^,^31^, there still remained some unresolved issues.

Recent efforts have been invested in studying the nature of the light-induced conical intersections (LICIs)^30^,^32^ which was first discussed for diatomics by applying Floquet representation. In a diatomic molecule, which has only one nuclear vibrational coordinate, it is not possible for two electronic states of the same symmetry to form a conical intersection (CI). However, this former statement is only true in field-free space. Conical intersections can be formed, even in diatomic molecules, if an additional degree of freedom e.g. rotation, is associated with the system exposed to strong laser fields. In this situation, the interaction of the transition dipole moment of the molecule with the external electric field leads to an effective torque toward the polarization direction of the light. By considering the rotational coordinate in the description as a dynamical variable, the change of nuclear dynamics due to the external light can be considered as arising from a LICI. The positions of these LICIs are determined by the laser frequency while the laser intensity controls the strength of the nonadiabatic coupling. Several theoretical and experimental works have demonstrated that the LICIs have strong impact on the spectroscopic and dynamical properties of molecules^31^,^33^,^34^,^35^,^36^,^37^,^38^,^39^,^40^,^41^,^42^,^43^,^44^,^45^,^46^,^47^,^48^.

There are many topical studies investigating the dissociation process of the 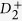 molecule within the LICI framework^37^,^38^,^39^,^40^,^41^. In these works it was found that using relatively small intensities (1 × 10^11^ to 1 × 10^12^ W/cm^2^) and propagating the dynamics of the 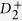 results in very small (practically zero) rates for the dissociation probabilities of the photofragments^39^ for certain vibrational eigenstates and photon energies. The dissociation yield still remains relatively small even at the highest studied intensity (1 × 10^14^ W/cm^2^). The obtained results are almost independent of the origins of the initial nuclear wave packet, which can start either from a vibrational eigenstate or a superposition of eigenstates, the Franck–Condon (FC) distribution, of the 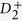.

Intensity dependence at fixed frequency regarding the vibrational trapping phenomenon in the 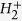 and 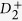 ions was intensively theoretically studied by Bandrauk and coworkers^5^,^6^,^7^,^8^,^27^. They highlighted that laser-induced avoided crossings provide a very important source of molecular stabilization and hence suppress dissociation at high intensities. They also found that when the diabatic (field-free) and the adiabatic (light-induced) vibrational levels coincide, the resulting resonances can minimize the photodissociation probabilities. Increasing the field intensities, trapping of the initial state into stable adiabatic states occurs due to the decreasing nonadiabatic couplings between the light-induced states. Their work resulted in a qualitatively correct picture of the vibrational trapping phenomenon mechanism.

In this article we investigate why, in certain situations, there is virtually no dissociation rate of the photofragments arising from the dissociation process of the 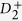 molecule. This paper expands previous investigations by trying to find a more accurate and quantitative connection between physical quantities including the coincidence of the diabatic and adiabatic vibrational levels, maximum overlaps between the diabatic and adiabatic wave functions and minima of the photodissociation probabilities. This is realized by calculating the difference between the diabatic and adiabatic vibrational energies, the overlap between the diabatic and adiabatic wave functions and the dissociation yield of the molecular photofragments over a wide range of photon energies (from *ω*_1_ = 24.799 eV, *λ*_1_ = 50 nm to *ω*_2_ = 3.100 eV, *λ*_2_ = 400 nm).

As was discussed in^39^,^40^, the bond hardening effect is highly dependent on the behavior of the system in low intensity field even if the actual applied intensity does not fall into this region. This is related to the fact that–not considering few cycle pulses–larger intensity pulses are built up gradually. Consequently, at the beginning of the pulse the role of the different adiabatic levels is determined by the low intensity behavior of the system. For this reason we use 1 × 10^11^ W/cm^2^ intensity in our simulations or even the zero intensity limit to determine the different features of the vibrational eigenstates of the adiabatic upper state. In the simulations we included only the two lowest lying electronic states of the 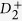
molecule. This decreases the validity of the results for the higher energy region—especially for the *λ* ≲ 100 *nm*—where the Rydberg states of the ion and even the doubly-ionized repulsive Coulomb states can play an essential role in the real processes. Fortunately, this inaccuracy in the description of the physical system does not invalidate our findings related to the bond hardening effect, which is widely studied within the two state approximation. One can consider that some of our results are valid only for a model system inspired by the 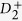 molecular ion.

## Theory and Methods

**Figure 1:**
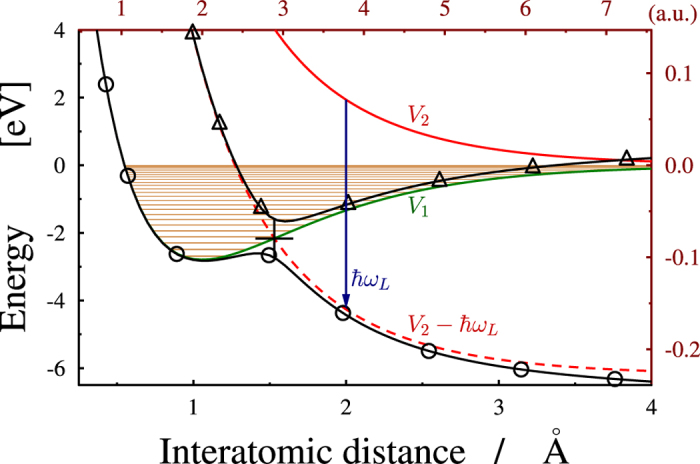
A cut through the potential energy surface of the 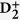 molecule as a function of inter atomic separation. Diabatic energies of the ground (*V*_1_) and the first excited (*V*_2_) states are displayed with solid green and red lines, respectively. The field dressed excited state (*V*_2_−*ħω*_*L*_; dashed red line) forms a light induced conical intersection (LICI) with the ground state. For the case of a laser frequency *ω*_*L*_ = 6.199 *eV* and field intensity of 3 × 10^13^(*W*)/(*cm*^2^) a cut through the adiabatic surfaces at *θ* = 0 (parallel to the field) is also shown by solid black lines marked with circles (*V*_*lower*_) and triangles (*V*_*upper*_). The position of the LICI is indicated with a cross.

Figure 1 shows the potential energy curves for 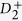 in dressed state representation. The electronic ground (*V*_1_(*R*) = 1*sσ*_*g*_) and the first excited (*V*_2_(*R*) = 2*pσ*_*u*_) eigenstates are considered as diabatic potentials and together, with the kinetic energy, form the field free Hamiltonian


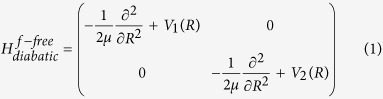


where *R* is the vibrational coordinate and *μ* is the reduced mass. The ion is excited by a resonant laser pulse from the *V*_1_(*R*) ground state to the repulsive *V*_2_(*R*) state. An electronic transition occurs due to the nonvanishing transition dipole moment and the corresponding 2 × 2 field dressed Hamiltonian matrix reads


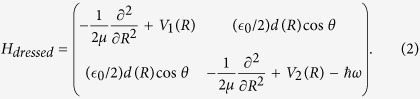


The off-diagonal elements of eq. (2) represent the radiative couplings, where 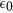 is the laser field amplitude, *ω* is the laser frequency which couples the two electronic states, *d*(*R*) is the transition dipole and *θ* is the angle between the polarization direction of the light and the direction of the molecular axes. The potential energies of *V*_1_(*R*) and *V*_2_(*R*) and the transition dipole moment were sourced from refs 49,50. The exact, time-dependent (TD) form of eq. (2), which is used extensively in the time-dependent calculations is also given here


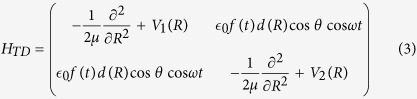


where *f*(*t*) is the envelope function of the laser pulse.

**Figure 2:**
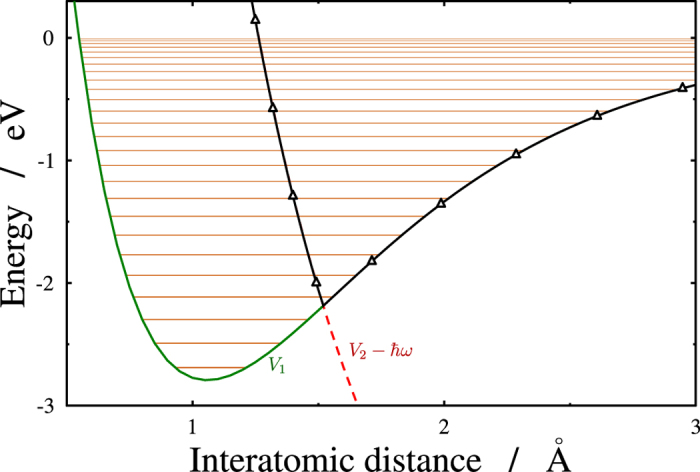
Diabatic and adiabatic cut of the potential energy surfaces along the inter atomic separation. Diabatic energies of the ground (*V*_1_; solid green) and the field dressed excited (*V*_2_ − *ħω*_*L*_; dashed red line) states are displayed. The adiabatic upper curve–calculated by using eq. (4) for the weak field limit–is denoted by solid black line marked with triangles. The different horizontal lines provide the appropriate diabatic (in orange) vibrational energy levels.

A convenient and approximate form for the adiabatic potential is assumed. The energy levels and the eigenfunctions of the upper adiabatic potential can easily be calculated for weak fields from a model potential (Fig. 2):


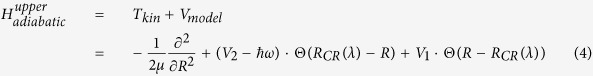


where Θ is the Heaviside step function, *λ* is the wavelength and *R*_*CR*_ is the crossing point between the ground *V*_1_(*R*) and the field dressed (*V*_2_(*R*) − *ħω*) excited states. This model potential in eq. (4) can be converted into the upper adiabatic potential at zero-field limit. It is now possible to calculate the vibrational energy levels (*E*_*ν*_) and wave functions (*ψ*_*ν*_) belonging to the different vibrational eigenstates of the diabatic potential in eq. (1) and the eigenvalues (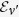) and eigenfunctions (*φ*_*ν*′_) of the upper adiabatic potential in eq. (4). A more detailed analysis requires new quantities derived from different combinations of the previously determined basic quantities. These are the difference of the adiabatic and diabatic eigenvalues


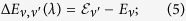


the adiabatic and diabatic eigenfunctions overlap,


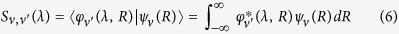


and the total dissociation probability:


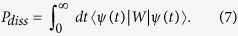


The Δ*E*_*ν*,*ν*′_(*λ*) and *S*_*ν*,*ν*′_(*λ*) were determined with a spacing of Δ*λ* = 1.0 in the interval of (50 nm–400 nm), −*iW* is the complex absorbing potential (CAP) applied at the last 5 a.u. of the grid related to the vibrational degree of freedom (*W* = 0.00005 · (*r* 70)^3^, if *r* > 70 a.u. on the 1*sσ*_*g*_ surface and *W* = 0.00236 · (*r* 75)^3^, if *r* > 75 a.u. on the 2*pσ*_*u*_ surface.

The multi-configuration time-dependent Hartree (MCTDH) method^51^,^52^,^53^,^54^,^55^ is used to solve the time-independent and time-dependent Schrödinger equations in conjunction with the eigenvalue and eigenfunction problems in the diabatic/adiabatic frameworks, (eqs (1 and 4)) and the dissociation dynamics (eq. (3)). Fast Fourier transformation-discrete variable representation (FFT-DVR)^56^ is used to characterize the vibrational degree of freedom, with *N*_*R*_ basis elements for the internuclear separation distributed within the range from 0.1 a.u. to 10.05 a.u. or 80 a.u. in the eigenstates or the dissociation yield calculations, respectively. These primitive basis sets (*χ*) are used to represent the wave function


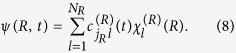


In the actual calculations, *N*_*R*_ = 256 is used for the eigenstates and *N*_*R*_ = 2048 for the dynamical calculations, respectively.

## Results and Discussion

Our present studies intensively probe the effect of molecular stabilization using accurate numerical calculations, performed in a wide range of energies. Detailed analysis is performed using a one-dimensional (1D) nuclear dynamical simulation and the initial nuclear wave packet is assumed to be in one of the vibrational eigenstates (*ν* = 0, 1, 2, 3, 4, 5, 6, 7, 8, 9) or Franck-Condon distribution of the vibrational states of the ion. The initial orientation of the molecules is assumed to be parallel to the external field in all cases. The dissociation rates for isotropic initial distribution can be approximated with large accuracy by dividing the results by 3 as this study is limited to low intensities. The energy interval (*ω*_1_ = 24.799 eV, *λ*_1_ = 50 nm to *ω*_2_ = 3.100 eV, *λ*_2_ = 400 nm) is chosen, so that it contains all the values belonging to the positions of the near zero dissociation probability of the studied vibrational levels. A linearly polarized Gaussian laser pulse, with intensity, *I*_0_ = 10^11^ *W*/*cm*^2^ and a pulse duration in full width at half-maximum (FWHM) *t*_*pulse*_ = 30 fs is used throughout the calculations.

Three physical properties, the difference of the diabatic and adiabatic eigenvalues (Δ*E*(*λ*)), the diabatic and adiabatic eigenfunctions’ overlap (*S*(*λ*)) and the dissociation probability (*P*_*diss*_) play an important role in the forthcoming analysis.

### Dissociation probability, wave function overlap and energy difference

The dissociation probability, eq. (7), is calculated in the chosen energy interval by maintaining a fixed light intensity. Wavelengths which result in a virtually zero dissociation probability from a particular vibrational energy level are sought, like in previous investigations^39^. Calculations have been performed for all chosen vibrational eigenstate, *ν* = 0, 1, …, 9, and the previously determined energy interval ensured that for each eigenstates all the wavelengths which belong to a minimum value of the dissociation probability were assigned. For example, nine different values for the wavelength were found for the *ν* = 9 vibrational level.

**Figure 3:**
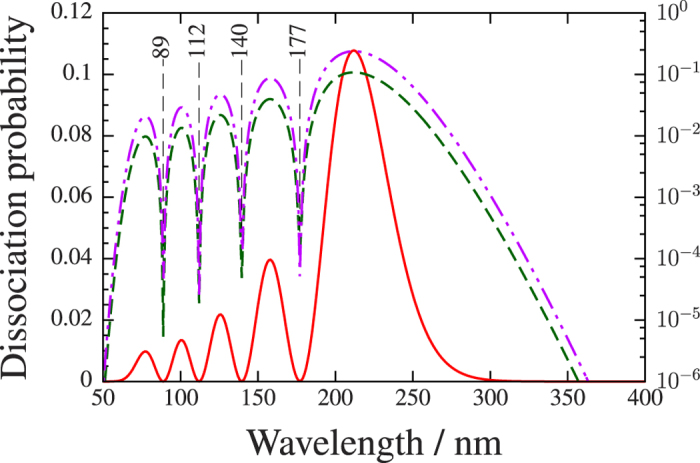
Total dissociation probabilities as a function of laser wavelength for the *ν* = 4 vibrational eigenstate. The applied energy/wavelength interval and the intensity are from *ħω*_1_ = 24.798 *eV*, *λ*_1_ = 50 *nm* to *ħω*_2_ = 3.0998 *eV*, *λ*_2_ = 400 *nm* and *I* = 1 × 10^11^(*W*)/(*cm*^2^), respectively. The total dissociation probability from the *ν* = 4 vibrational eigenstate is displayed by the solid (linear scale on the left side) and dashed (logarithmic scale on the right side) curves. The dashed–dotted curve displays (logarithmic scale on the right side) the differential dissociation rate with kinetic energy of fragments being *E*_*ν*_ + *ħω* when the simulation was started from Franck–Condon distribution.

Figure 3 shows the results for the *ν* = 4 vibrational level for the case when the initial nuclear wave packet starts from (i) a vibrational eigenstate or from (ii) a superposition of eigenstates, the Franck–Condon, (FC) distribution, of the 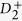. The dissociation rate has four minima at the *λ*_*D*_(4, 3) = 88.8 nm, *λ*_*D*_(4, 2) = 112 nm, *λ*_*D*_(4,1) = 139.5 nm and *λ*_*D*_(4, 0) = 177.1 nm wavelengths. Simulations in which the wave packets start from a vibrational eigenstate use pulses with a 30 fs duration centered around 0 fs and the total dissociation probabilities are calculated. In the superposition situation, pulses with a 30 fs duration are applied. These pulses are centered around 34 fs during which time approximately one and a half vibration cycle takes place on the diabatic lower surface *V*_1_^38^. The yield of total dissociation probability does not provide any information about the dissociation rate resulting from a particular vibrational state because all of the vibrational eigenstates are included in the initial wave packet. Thus, attention turned to the kinetic energy (KER) release spectra and the dissociation rate at the *E*_*ν*_ + *ħω* energy was studied.

Results for the dissociation yield obtained by assuming FC distribution of the nuclear wave packet are surprisingly similar to those obtained from initiating the dynamics from one of the vibrational eigenstates of the Hamiltonian. This similarity is related to the fact that, on one hand the applied laser pulse is long enough to resolve properly the vibrational energy eigenstates and on the other hand its intensity is in the linear respond regime. These two features allow us to conclude that the structure of the initial wave packet does not significantly affect the underlying physical mechanism in the bond hardening effect. (Not displayed results for other vibrational states clearly confirm this statement, as well). For the sake of simpler analysis initial wave packets assuming vibrational eigenstate form are used in the further calculations.

**Figure 4:**
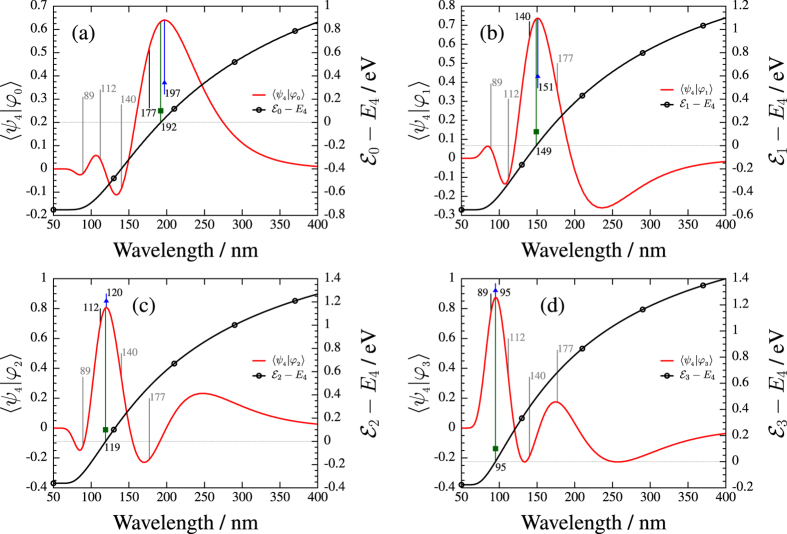
Overlap *S*_*ν*,*ν*′_(*λ*) between the diabatic (*ψ*_*ν*_; *ν* = 4) and adiabatic (*φ*_*ν*′_) eigenfunctions and the difference of the diabatic and adiabatic eigenvalues (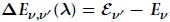; *ν* = 4) as a function of wavelength. The value of *S*_*ν*,*ν*′_(*λ*) (solid curve) is given by the scale on the left side, while the value of Δ*E*_*ν*,*ν*′_(*λ*) (dotted curve) is given by the scale on the right side. Bars denote the wavelengths for which the total dissociation probability is minimal. Bars with triangle and bars with square present the *λ*_*O*_(*ν*, *ν*′) and *λ*_*E*_(*ν*, *ν*′) wavelengths corresponding to the maximum value of *S*_*ν*,*ν*′_(*λ*) and the zero value of Δ*E*_*ν*,*ν*′_(*λ*), respectively. In panel (**a**) (*ν*′ = 0), in panel (**b**) (*ν*′ = 1), in panel (**c**) (*ν*′ = 2) and in panel (**d**): (*ν*′ = 3).

The next task was to obtain the diabatic and adiabatic eigenfunctions’ overlap which was calculated using eq (6). For each diabatic vibrational level (*ν*) *ν*′ = 0, 1, … *ν* − 1, adiabatic eigenstates and correspondingly *ν* different overlap functions exist. Figure 4 shows these functions for the *ν* = 4 vibrational level. The 4 different panels belong to the corresponding values of *ν*′ from 0 to *ν* − 1, and the obtained curves are scaled on the left side of the panels.

**Figure 5:**
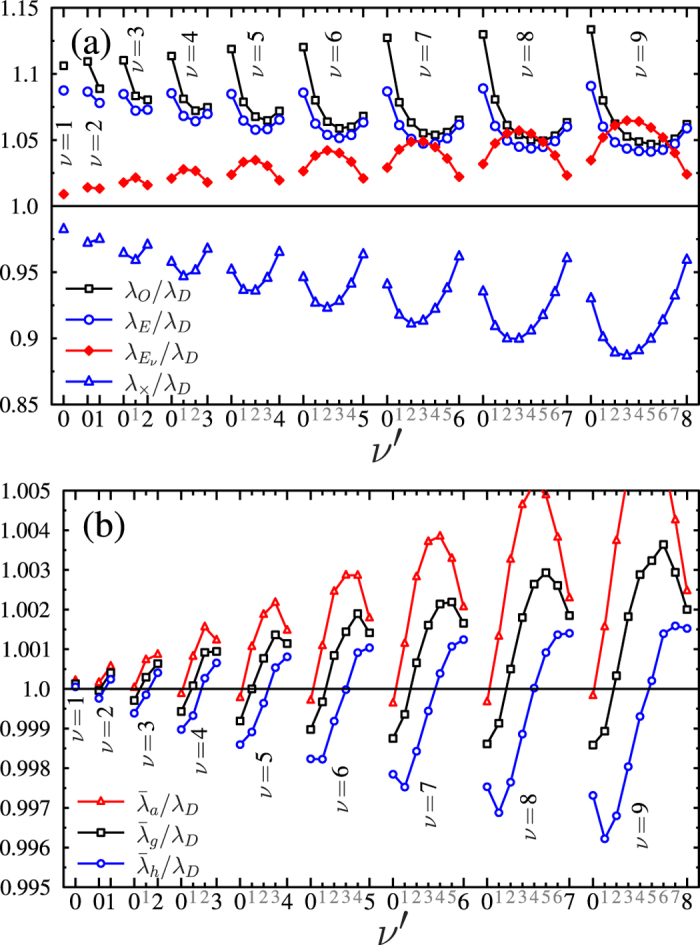
Ratios of the different wavelengths with respect to the minimum value of the dissociation probability *λ*_*D*_. Panel (**a**): *λ*_*O*_ is the wavelength belonging to the maximum value of the wave functions’ overlap, *λ*_*E*_ is the wavelength where the adiabatic and diabatic eigenvalues are the same, 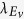 is the wavelength according to eq. (9) and *λ*_×_ is the wavelength according to eq. (10); Panel (**b**): 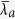, 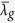 and 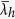 are the weighted arithmetic, geometric and harmonic means of 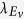 and *λ*_×_ as defined in eqs (11–13).

The difference of the adiabatic and diabatic eigenvalues eq (5), is determined and *ν* energy difference curves are obtained for each value of *ν*. Figure 4 shows these curves which are scaled on the right side of the panels. It can be expected, based on previous theoretical studies^8^,^9^,^27^ that, for a given pair of diabatic and adiabatic eigenstates with the feature of *ν* > *ν*′, both the maxima of the overlap between the diabatic and adiabatic vibrational wave functions at *λ* = *λ*_*O*_(*ν*, *ν*′) and the matching of the diabatic and adiabatic eigenvalues at *λ* = *λ*_*E*_(*ν*, *ν*′) are closely related to the minimal dissociation yield or bond hardening situation, which takes place at special photon energies *λ* = *λ*_*D*_(*ν*, *ν*′). These special values of the wavelength are presented in the first four columns of Table 1 for all the studied vibration levels (*ν* = 0, 1, …, 9). If the values of these three wavelengths (*λ*_*D*_, *λ*_*O*_ and *λ*_*E*_) are close to each other for a certain pair of vibrational frequency (*ν*, *ν*′), the values of the (*λ*_*O*_)/(*λ*_*D*_) and (*λ*_*E*_)/(*λ*_*D*_) ratios are also close to 1. However, Fig. 5 shows that this is not the case as the (*λ*_*O*_)/(*λ*_*D*_) and (*λ*_*E*_)/(*λ*_*D*_) wavelength rates systematically deviate from 1 within a range of 5–15% for all cases. The significantly large differences between the wavelengths suggest that the physics behind the trapping effect may not be described adequately by studying the overlap of the wave functions or energy differences and it is expected, that some other factors may play a role.

### The role of the nodal structure

**Figure 6:**
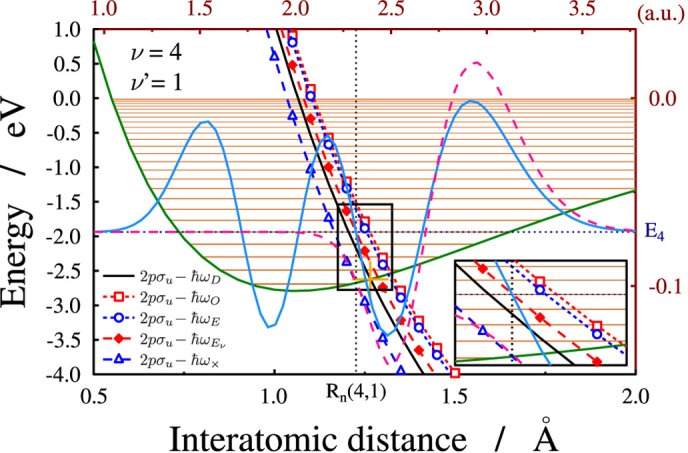
The *ψ*_*ν*_(*ν* = 4) diabatic eigenstate (cyan (light gray) line) and 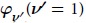 adiabatic eigenstate (dashed pink curve) for the case of maximal overlap (*λ*_*O*_(4, 1)) with the diabatic *ν* = 4 eigenstate. The zero line for the wave functions is placed to the energy level of the *ν* = 4 vibrational state. The *V*_1_ diabatic and *V*_2_ potential energies with five field-dressed excited states are also shown. The solid black line denotes the shift by a laser field with the wavelength *λ*_*D*_(4, 1) (*ω*_*D*_(4, 1) frequency), for which the dissociation of the 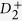 is minimal. Lines marked with empty squares and circles are shifted by the energies of *ħω*_*O*_ and *ħω*_*E*_, respectively. The line marked with solid diamonds corresponds to a shift with 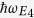, so that the energy of the dressed state at the third node (*R*_*n*_(4, 1)) of the *ν*_4_ diabatic eigenfunction is equal to the *E*_4_ vibrational eigen energy. The *ħω*_×_ is the photon energy (*λ*_×_ (4, 1) wavelength) by which shifting the *V*_2_ state (marked with triangles) a crossing with the ground state potential is formed at the position of the *R*_*n*_(4, 1) node. The inset in the bottom right corner displays a zoomed version of the central area.

This section starts with a simple analysis of the adiabatic and diabatic wave functions overlap. It is obvious that the node positions of adiabatic wave function should be close to the node positions of the diabatic function in order to obtain a significantly large overlap. The adiabatic wave function fades to zero at such internuclear distances where the adiabatic potential is above the adiabatic energy level. Applying a photon energy which corresponds to the dissociation minimum results in the energies of the diabatic and adiabatic states being similar. In such a case, the disappearance of the adiabatic wave function can be expected to approximately take place where the adiabatic potential is equal to the energy of the diabatic eigenstate. Thus, it can be assumed that for a large overlap the matching of the adiabatic potential to the diabatic energy level should take place around one of the nodes of the diabatic eigenfunction. The wavelength 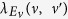 is where the matching of the adiabatic potential to the energy of the diabatic eigenstate takes place at the (*ν* − *ν*′)^*th*^ node (*R*_*n*_(*ν*, *ν*′)) of the diabatic wave function, i.e., after which there are additional *ν*′ nodes (Fig. 6):


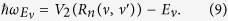


There is another possible way to demonstrate the importance of the nodes of the nuclear wave packet in the process of molecular stabilization. In the light-induced picture of dissociation, the nonadiabatic coupling between the two surfaces will be the largest around the crossing of the ground and the dressed excited state diabatic potentials. Therefore, a reasonable reduction in the dissociation yield can be expected if the initial diabatic wave packet has a node at, or close to this crossing. To check the validity of this approach, the wavelengths for which the crossing of the potentials takes place at one of the nodes of the diabatic wave function was determined. These values are denoted as *λ*_×_ (*ν*, *ν*′) and the corresponding photon energies can be calculated as


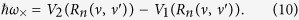


Table 1 shows both of these newly introduced quantities and the positions of their dependant nodes.

The accuracy of the above constructed approach was checked by calculating the ratios with *λ*_*D*_ and the obtained (*λ*_×_)/(*λ*_*D*_) and 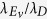 rates are shown in Fig. 5a. For lower lying vibrational states, both these two new formulas, based on the position of the nodes, provide a better forecast of the wavelength of the minimal dissociation yield than the preliminary defined *λ*_*E*_ or *λ*_*O*_ values. *λ*_×_ underestimates *λ*_*D*_ approximately twice as much as 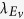 overestimates. This observation can be used to construct some additional semiempirical formulas which might balance these two opposite deviations in the *λ*_×_ and 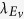
wavelengths (see in Fig. 5a). In these formulas the 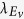 wavelength always takes part with twice the weight in. Obtained combinations are: the weighted arithmetic, geometric and harmonic means (arithmetic mean of the photon energies) of the former two node based approximations for the position of the minimal dissociation rate:


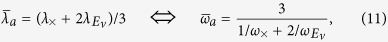



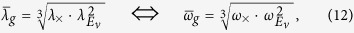



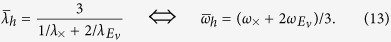


The ratios of the above constructed mixed quantities to the dissociation minimum (*λ*_*D*_) are shown in Fig. 5b. All the three formulas lead to very similar results predicting the dissociation minima with less than a half percent error, except for a few cases. The harmonic mean, defined in eq. (13), is the most balanced combination as its values remain in the narrowest interval around *λ*_*D*_. The fact that these wavelengths, especially their special combination provide an almost perfect molecular stability, implies that the nodes of the nuclear vibrational wave packet play an essential role in the bond hardening effect.

The above numerical results support the theoretical expectations: initiating the nuclear dissociation dynamics from a given vibrational level, one can always find the appropriate photon energy that minimizes the dissociation yield. The latter holds for the total dissociation probability when the initial wave packet starts from one of the vibrational eigenstates and for the kinetic energy release spectra (KER) at a certain (*E*_*ν*_ + *ħω*) energy when the initial wave packet starts from a FC distribution of ionic vibrational eigenstates. At the heart of this work are the nodes of the nuclear vibrational wave packet which can quantitatively be associated with the bond hardening effect.

Our showcase example throughout this study was the 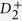 molecule. One may assume that the present findings are independent from 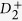 and are valid for any other molecular systems, as well.

## Conclusions

In this paper, a series of numerical calculations have been performed, over a wide range of photon energies in order to provide accurate results for the vibrational trapping phenomenon. It was demonstrated that the nodal structure of the nuclear vibrational wave packets have an important role in the molecular stabilization process. We have found an accurate quantitative connection between the nodes of the nuclear vibrational wave packet and the minimum of the dissociation rate. In contrast to the literature, the importance of the nodes of the vibrational wave packet is strongly stressed because an almost perfect molecular stabilization can be obtained using our recently proposed formula (eq. (13)) of the node based wavelength.

However, we have yet to clearly identify the background mechanism behind the harmonic mean combination of the 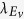 and *λ*_×_ wavelengths, which can provide the best description of the phenomena. Our studies will continue and will be extended in this direction and we expect to form a better understanding about the complex physical phenomena beyond the vibrational trapping effect. Nevertheless, we hope that the present findings will stimulate experimental works in the near future.

## Additional Information

**How to cite this article**: Badankó, P. *et al*. Molecular vibrational trapping revisited: a case study with D2+. *Sci. Rep.* **6**, 31871; doi: 10.1038/srep31871 (2016).

## Figures and Tables

**Table 1:** Characteristic *λ* wavelengths corresponding to the *ν*, *ν*′ (*ν*′ < *ν*) different vibrational diabatic and adiabatic levels.

*ν*,*ν*′	*λ*_*D*_(*nm*)	*λ*_*O*_(*nm*)	*λ*_*E*_(*nm*)	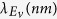	*λ*_×_ (*nm*)	*R*_*n*_(*au*)	*ν*,*ν*′	*λ*_*D*_(*nm*)	*λ*_*O*_(*nm*)	*λ*_*E*_(*nm*)	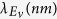	*λ*_×_ (*nm*)	*R*_*n*_(*au*)
1,0	111.21	123.02	120.94	112.21	109.27	2.05	7,0	258.66	291.60	281.10	266.19	243.32	3.16
							7,1	206.70	222.93	219.37	215.55	189.71	2.82
2,0	133.27	147.85	144.81	135.15	129.57	2.29	7,2	170.00	180.76	178.66	178.28	154.89	2.54
2,1	100.21	109.11	108.03	101.54	97.72	1.90	7,3	141.47	149.29	148.16	148.39	129.20	2.29
							7,4	118.09	124.45	123.67	123.37	108.91	2.05
3,0	154.67	171.72	167.78	157.42	149.17	2.48	7,5	98.08	103.57	103.11	101.62	91.97	1.81
3,1	120.08	130.09	128.75	122.67	115.18	2.13	7,6	79.78	84.98	84.69	81.55	76.74	1.56
3,2	93.54	101.06	100.36	95.03	90.80	1.80							
							8,0	293.15	331.23	319.24	302.48	274.19	3.33
4,0	177.09	197.19	192.21	180.79	169.62	2.66	8,1	234.31	253.22	248.51	245.42	213.03	2.98
4,1	139.53	150.84	149.05	143.41	132.12	2.32	8,2	193.05	204.87	202.61	203.66	173.72	2.70
4,2	111.96	120.04	119.15	114.94	106.51	2.02	8,3	161.23	169.95	168.48	170.44	145.06	2.45
4,3	88.78	95.42	94.97	90.38	85.91	1.72	8,4	135.49	142.27	141.41	142.92	122.74	2.21
							8,5	113.90	119.54	119.00	119.44	104.50	1.99
5,0	201.42	225.35	218.50	206.20	191.71	2.83	8,6	95.12	100.18	99.79	98.76	88.92	1.77
5,1	159.95	172.56	170.31	165.29	149.80	2.49	8,7	77.74	82.67	82.41	79.55	74.67	1.52
5,2	130.10	138.91	137.62	134.62	121.78	2.20							
5,3	106.14	113.02	112.32	109.37	100.37	1.93	9,0	332.84	377.34	363.08	344.38	309.60	3.50
5,4	85.13	91.25	90.69	86.79	82.17	1.66	9,1	265.72	286.96	281.69	279.52	239.35	3.14
							9,2	218.95	232.59	229.52	232.31	194.68	2.85
6,0	228.36	255.84	247.97	234.41	216.07	3.00	9,3	183.16	192.80	191.14	195.02	162.45	2.60
6,1	182.13	196.72	193.49	189.09	168.81	2.66	9,4	154.46	162.00	160.89	164.36	137.60	2.37
6,2	149.22	158.75	157.26	155.50	137.74	2.37	9,5	130.69	136.82	136.08	138.46	117.57	2.15
6,3	123.32	130.57	129.67	128.27	114.47	2.12	9,6	110.45	115.65	115.15	116.18	100.90	1.94
6,4	101.67	107.78	107.14	105.09	95.71	1.87	9,7	92.64	97.36	97.00	96.36	86.37	1.73
6,5	82.20	87.80	87.40	83.92	79.19	1.61	9,8	76.00	80.70	80.47	77.82	72.91	1.49

*R*_*n*_ denotes the positions of the nodes of *ψ*_*ν*_ diabatic wave functions.
